# Precision Nutrition and Cardiovascular Disease Risk Reduction: the Promise of High-Density Lipoproteins

**DOI:** 10.1007/s11883-023-01148-5

**Published:** 2023-09-13

**Authors:** Brian V. Hong, Joanne K. Agus, Xinyu Tang, Jack Jingyuan Zheng, Eduardo Z. Romo, Susan Lei, Angela M. Zivkovic

**Affiliations:** Department of Nutrition, University of California, Davis, Davis, CA 95616 USA

**Keywords:** Precision nutrition, Cardiometabolic health, Cardiovascular disease, Personalized nutrition

## Abstract

**Purpose of Review:**

Emerging evidence supports the promise of precision nutritional approaches for cardiovascular disease (CVD) prevention. Here, we discuss current findings from precision nutrition trials and studies reporting substantial inter-individual variability in responses to diets and dietary components relevant to CVD outcomes. We highlight examples where early precision nutrition research already points to actionable intervention targets tailored to an individual’s biology and lifestyle. Finally, we make the case for high-density lipoproteins (HDL) as a compelling next generation target for precision nutrition aimed at CVD prevention. HDL possesses complex structural features including diverse protein components, lipids, size distribution, extensive glycosylation, and interacts with the gut microbiome, all of which influence HDL’s anti-inflammatory, antioxidant, and cholesterol efflux properties. Elucidating the nuances of HDL structure and function at an individual level may unlock personalized dietary and lifestyle strategies to optimize HDL-mediated atheroprotection and reduce CVD risk.

**Recent Findings:**

Recent human studies have demonstrated that HDL particles are key players in the reduction of CVD risk. Our review highlights the role of HDL and the importance of personalized therapeutic approaches to improve their potential for reducing CVD risk. Factors such as diet, genetics, glycosylation, and gut microbiome interactions can modulate HDL structure and function at the individual level. We emphasize that fractionating HDL into size-based subclasses and measuring particle concentration are necessary to understand HDL biology and for developing the next generation of diagnostics and biomarkers. These discoveries underscore the need to move beyond a one-size-fits-all approach to HDL management. Precision nutrition strategies that account for personalized metabolic, genetic, and lifestyle data hold promise for optimizing HDL therapies and function to mitigate CVD risk more potently.

**Summary:**

While human studies show HDL play a key role in reducing CVD risk, recent findings indicate that factors such as diet, genetics, glycosylation, and gut microbes modulate HDL function at the individual level, underscoring the need for precision nutrition strategies that account for personalized variability to optimize HDL’s potential for mitigating CVD risk.

## Introduction

Although the concept of “personalized nutrition” or “individualized nutrition” is not new [1, 2], it was not until recently that “precision nutrition” became more widely accepted and appreciated as an important strategy for improving cardiovascular disease (CVD) risk reduction [3, 4]. An astounding proportion of CVD risk is attributable to diet and lifestyle factors. Meta-analysis has found that individuals with the healthiest lifestyle behaviors had up to 71% lower cardiovascular risks compared to those with unhealthy lifestyles, with consistent associations across diverse populations, indicating that healthy lifestyles could reduce the global CVD burden [5]. Yet diet and lifestyle interventions are notoriously difficult for individuals to implement, with low adherence in the long-term [6] standing in the way of realizing the full potential of diet and lifestyle management to prevent CVD. The lack of adherence to preventive diet interventions has a number of plausible explanations, including obstacles such as access to and cost of food, as well as time, knowledge, and other resources needed to shop for and prepare healthier meals [7]. An additional factor is the array of confusing and conflicting information that is pervasive throughout the nutrition literature. However, the reasons for this conflict are not simply that nutrition studies are difficult to plan and execute, that human participants do not adhere to study protocols, or that nutrition science lacks rigor, as many have asserted. Increasing or decreasing a particular dietary component or following a particular diet and lifestyle intervention inherently has differential effects on different individuals in different contexts.

The current approach to dietary recommendations for CVD prevention has been to assess interventional and epidemiological studies and arrive at a consensus population-wide recommendation based on the strength of the evidence [8]. While this seems like a reasonable approach, the fundamental issue is that people are different not just from each other but from themselves at different points in time, and thus population-based dietary recommendations, in effect, simply do not necessarily work at the individual level. Any given patient has their own specific genetic predispositions, current metabolic phenotype, health status, lifestyle factors, health history, and life circumstances which dictate whether any given dietary component should be increased, decreased, or avoided entirely to optimize their risk profile [1].

Many researchers advise that the next step toward precision nutrition from population-level recommendations are recommendations for more narrowly defined populations [9]. However, the process of defining these narrowly defined populations will inevitably lead to the same general obstacle: that no matter how narrowly we define a population (e.g., post-menopausal, African-American female with metabolic syndrome) there will still be inter-individual variability in response that cannot be explained by the defining characteristics used to categorize an individual into a population group. For example, the post-menopausal African-American female with metabolic syndrome may have either low or adequate status for any of the micronutrients, she may or may not have high Lp(a), low muscle mass, low bone mineral density, sensitivity to gluten, high C reactive protein, etc. The more narrowly we define the population group of interest the more we realize that there are as many population groups as there are individuals. Instead, we contend that the more successful approach is to discover specific targets for intervention where precision nutrition can be implemented to modify risk.

In contrast to population-level recommendations that primarily target disease prevention for public health, evidence-based practice involves clinicians using the best available research while considering professional expertise, disease mechanisms, and patient characteristics, aligning with individualized care [10]. Precision nutrition techniques can further help personalize dietary advice by providing additional genetic variants, epigenetic factors, microbiome, and detailed monitoring of individual responses to foods. By complementing population-level guidelines with precision nutrition strategies, clinicians can provide tailored dietary recommendations aimed at maximizing benefit and minimizing risk for each individual. For example, in patients with hypertension, precision nutrition techniques may identify individuals with epigenetic markers or gut microbial profiles associated with salt sensitivity [11–13]. Dietary guidance could then be individualized accordingly, leading to improved efficacy and therapeutic benefit. In this way, precision nutrition techniques hold promise for assisting clinicians in translating general dietary guidelines into more personalized interventions.

In this review article, we discuss the current evidence from precision nutrition trials and recent studies reporting on inter-individual variability in response to diets and dietary components relevant to CVD prevention, discuss examples of dietary components where early precision nutrition research points to actionable intervention targets, and discuss the potential of high-density lipoproteins (HDL) as a next generation suite of precision nutrition targets for CVD prevention.

## Evidence from Precision Nutrition Trials and Studies Reporting Inter-Individual Variability in Response to Diets and Dietary Components

In the past decade, large intervention studies have revealed considerable inter-individual variability in responses to foods and diets [14–17]. These studies built on previous work from previous generations [18–20] investigating heterogenous responses to dietary interventions. Of particular interest for CVD risk management are outcomes related to plasma lipids, blood pressure, and inflammation. The study of the effects of different diets and dietary components, particularly dietary fats (i.e., monounsaturated vs. polyunsaturated vs. saturated fatty acids) on plasma lipid profiles, blood pressure, and inflammation has a long history and has been reviewed elsewhere [21, 22]. Below we focus on evidence from recent studies reporting on inter-individual variability in the effects of dietary interventions on these three key sets of outcomes in terms of their direct applicability to the management of CVD risk.

### Plasma Lipids

Plasma lipids have been shown to be an important determinant of systemic inflammation and play a key role in modulating the postprandial inflammatory response and subsequent CVD risk. For example, in a clinical intervention study investigating the consumption of a standardized high-fat meal in 1002 adults from the PREDICT 1 trial, postprandial plasma triglycerides and visceral fat were key predictors of plasma GlycA [15], which is linked to systemic inflammation, CVD risk, and mortality [23, 24]. Furthermore, in a randomized controlled trial of 92 men and women with abdominal obesity and low HDL cholesterol, the differential effects of saturated fatty acids from cheese versus butter on LDL cholesterol may have important implications for inflammation and cardiometabolic disease risk, especially in those with already high LDL cholesterol [25]. Notably, the impact of saturated fatty acids from butter versus cheese on LDL cholesterol depended on baseline LDL levels in which the increase in LDL cholesterol was much greater with butter compared to cheese, but only in those with high baseline LDL cholesterol [25]. Together, these findings highlight the key role of plasma lipids in modulating postprandial inflammation and the importance of individual plasma lipid levels in influencing the variability in response to different sources of dietary fats, which can subsequently impact CVD risk markers.

Another player associated with dietary response is apolipoprotein E (ApoE). ApoE is a major HDL-associated protein responsible for transporting lipids including cholesterol in the bloodstream [26]. Its genetic polymorphism, particularly the E4 allele, has been shown to be associated with chronic diseases, including CVD, type II diabetes, and Alzheimer’s disease [27, 28]. Due to this link between *APOE* and disease risk, genetic factors such as *APOE* genotype and polymorphism can influence lipid metabolism in response to dietary changes. For example, in a retrospective analysis of the DIVAS study involving 120 participants at moderate CVD risk, researchers investigated the replacement of saturated fatty acids with monounsaturated and n-6 polyunsaturated fatty acids [29]. They discovered that individuals with “TT” homozygous allele at the *APOE* single nucleotide polymorphism (SNP) rs1064725 (90% of the population) were more responsive to dietary fat compositional changes, showing reduced total plasma cholesterol after substituting saturated fats with monounsaturated fats, but not n-6 polyunsaturated fatty acids, for 16 weeks. Interestingly, this SNP was not associated with changes in HDL-C or LDL-C [29]. Additionally, the SATgenε study of 88 normolipidemic *APOE3/E3* (*n* = 44) or *APOE3/E4* (*n* = 44) participants revealed a significant gene × diet interaction in which the *APOE* genotype influenced plasma triglycerides and inflammation due to dietary fat changes [30]. *APOE3/E4* carriers exhibited a greater lowering of triglycerides with high-fat intake supplemented with 3.45 g docosahexaenoic acid (30% vs. 17%) and increased C-reactive protein (CRP) with high saturated fat intake compared to *APOE3/E3* carriers, whereas no significant interaction between *APOE* genotype × diet was observed for LDL-C and HDL-C [30].

Similarly, the RISCK study cohort of 389 adults revealed a significant gene-diet interaction where *APOE4* carriers exhibited greater reductions in plasma total cholesterol and apolipoprotein B (ApoB) compared to *APOE3* homozygotes when saturated fat was replaced with a low fat, low glycemic index carbohydrate diet over 24 weeks [17], which may be attributed to higher baseline total cholesterol and ApoB concentrations in *APOE*4 carriers compared with *APOE3* homozygotes and *E2* carriers. This indicates that *APOE4* carriers may exhibit a differential plasma lipid response compared to *APOE3* homozygotes when dietary fat intake is altered. Building on these findings, other researchers have implemented genotype-based personalized nutrition interventions. For example, in the Food4Me study of 1466 adults, *APOE4* carriers had higher baseline total cholesterol and showed greater reductions in total fat and saturated fat intake after 6 months of receiving gene-based personalized nutrition advice compared to standard dietary advice, which suggests that adherence to gene-based dietary recommendations for limiting their saturated fat intake may be more effective in those with higher genetic risk [31]. Collectively, these studies suggest that personalized dietary recommendations based on *APOE* genotype may optimize CVD risk reduction in certain high-risk subgroups.

### Blood Pressure

Hypertension, characterized by an increase in blood pressure, is a significant risk factor for CVD [32, 33]. The condition is often caused by the narrowing of blood vessels and an increase in blood volume. There are two main categories of hypertension based on their etiology—primary and secondary. According to the 2017 ACC/AHA hypertension guidelines, secondary hypertension is linked to an underlying and identifiable condition, dietary factors, or medications [32]. On the other hand, primary hypertension is characterized by a gradual and systemic increase in blood pressure due to the progression of atherosclerosis influenced by factors like obesity, diet, and stress [32]. In reality, many individuals experience hypertension attributed to both types, and dietary and lifestyle modification have been shown to be effective at reducing these risks. In this section, we will explore inter-individual variability in response to dietary changes as it relates to the etiologies of hypertension.

One of the most extensively studied dietary components concerning hypertension is sodium [34], which is primarily found in dietary salt. High sodium consumption leads to water retention by the kidneys and expands blood volume [35]. Genetic variations or disease-related factors that influence the function of the renal sodium pump, such as genes affecting alpha aductins or renal failure, can increase or decrease the effect of sodium on blood pressure [35]. Moreover, Ying and Sanders [36] discovered that dietary sodium can modulate arterial wall gene expression, affecting nitrous oxide synthesis, and altering arterial dilation capacity in response to sodium [37]. Polymorphism in the G-to-T nucleotide at position 894 (G894T) of the endothelial nitric oxide synthase (eNOS) has been shown to play a role in the differences in hypertension risk between different ethnicities [37]. Additionally, salt-sensitivity can influence sodium consumption by affecting salt-appetite [34]. Generally, population studies have indicated that reducing dietary sodium overall can lower blood pressure [34]. However, a study by Sacks et al. [38] demonstrated that the Dietary Approaches to Stop Hypertension (DASH) diet is particularly effective at reducing blood pressure in certain populations, such as those with existing hypertension, black individuals, and women.

### Inflammation

Biomarkers, such as interleukin-6 (IL-6), tumor necrosis factor-alpha (TNF-a), and CRP, are commonly used to determine chronic inflammation and are often associated with the risk of CVD [39, 40]. The Mediterranean diet has been proven effective in reducing these inflammatory markers [40]. However, it remains unclear who can benefit the most from this diet and whether specific components are crucial for its anti-inflammatory properties. In this context, we will highlight the differences in how omega-3 and fiber affect responders and non-responders, as these two dietary components are often linked to their ability to lower inflammation.

Omega-3 supplementation has been demonstrated to lower inflammatory cytokines and arterial adhesion molecules [41, 42]. Omega-3 alpha linolenic acid (ALA) is commonly found in non-animal sources like chia and flax seeds, and it needs to be processed by elongase and desaturase enzymes to the long-chain anti-inflammatory fatty acids eicosapentaenoic acid (EPA) and docosahexaenoic acid (DHA). However, generally speaking, most people have very low conversion of ALA to EPA and DHA [43], and thus, many individuals require direct sources of EPA and DHA, such as salmon and krill [41, 42]. However, even when taking EPA and DHA directly, there is a high degree of variability in response, with large differences in magnitude of change in both the EPA and DHA themselves, as well as their oxylipin derivatives [18, 44, 45]. Recent studies have explored genetic variations to explain the responder and non-responder effect [46]. One such study evaluated genetic risk scores (GRS) in two cohorts: the Fatty Acid Sensory study (FAS) and the European FINGEN study, and it identified 31 SNPs associated with the efficacy of omega-3 supplementation in reducing triglyceride levels [46]. Previous research has also shown responder differences in those carrying FADS1 and FADS2 genes, using genome-wide association studies (GWAS) analysis for other omega-3 responder genes [47, 48]. Additionally, a meta-analysis revealed that plasma omega-3 concentration is negatively associated with TNF-a, IL-6, and CRP concentrations, especially in non-diseased individuals with a BMI below 30, but this association weakens in those with a BMI over 30 [39]. Thus, both genetics and metabolic/physiologic state affect response to omega-3 fatty acids and determine their effectiveness in reducing inflammation.

Fiber is another dietary factor that has been studied for its potential to lower inflammation [49, 50]. When comparing the high-fiber Dietary Approach to Stop Hypertension (DASH) and fiber-supplemented (psyllium) diet in hypertensive subjects with obesity and normotensive lean subjects, both diets showed significant reductions in CRP, with the fiber-supplemented diet having a greater effect on normotensive lean subjects but not on hypertensive subjects with obesity [49]. The study suggests that the fiber amount for hypertensive subjects with obesity may need to be increased, or the CRP-lowering effect of fiber may be more effective if combined with other treatments for those with insulin resistance and metabolic syndrome [49]. Recent studies have further linked the efficacy of fiber in reducing CRP to the composition of the microbiome [50]. The study revealed that the consumption of fiber is most effective in reducing CRP for subjects with a low *Prevotella copri* population, while those already with *P. copri* did not experience a reduction in CRP regardless of fiber consumption [50]. Although some aspects of the response to changes in dietary fiber content are generally universal (e.g., increased fiber-fermenting microbes and decreased protein- and lipid-degrading microbes on high-fiber diets [51, 52]), others are highly unique to the individual’s baseline microbiome. For example, the colitis-inducing, pro-inflammatory, bile-degrading microbe *Bilophila wadsworthia* was increased in response to a low-fiber, high-fat fast food diet only in those individuals who harbored *B. wadsworthia* at baseline and in those for whom saturated fat intake was lower prior to the change to the fast food diet [51]. These findings highlight that an individual’s long-term diet and gut microbiome profiles have an important effect on their response to short-term dietary changes, and that context is crucial in determining one’s response to any particular diet or dietary ingredient.

## Examples of Dietary Components with Actionable Intervention Targets

Nutrigenetics has become an integral part of precision nutrition as it allows diet to be tailored based on genetic variability that influences nutritional needs and responses. The most established nutrigenetic studies focus on SNPs that directly affect nutrient metabolism. GWAS are particularly useful in identifying strong genetic associations with metabolic states and SNP genes, especially in rare monogenic disorders [53, 54]. For example, monogenic disorders like familial hypercholesterolemia (FH) lead to elevated LDL cholesterol due to mutations in the LDL pathway [55, 56]. Treatment for FH primarily involves a combination of statins and other pharmacological approaches from an early age, while nutritional interventions, such as reducing total lipid and cholesterol consumption, and exercise are typically prescribed as complementary therapies [56].

In contrast, most gene variations associated with CVD are polygenic in nature, involving multiple variants. For instance, GWAS identified variants in the intron region of the fat mass and obesity-associated (FTO) gene, initially linked to type 2 diabetes, and later associated with obesity [57], metabolic syndrome [54], and subsequently, CVD [58]. FTO is a feeding-signal-associated gene, influencing an individual’s overall food intake and energy homeostasis [57], contributing to the etiology of metabolic syndrome, a risk factor for CVD, when coupled with poor diet and lifestyle behaviors [54]. As more gene sequencing data emerges, the relationship between FTO genes and obesity seems to hold true for certain populations [59] but not others [60], and its manifestation also appears to be dependent on other diet and lifestyle factors [61], such as exercise [62]. In this section, we will discuss two diet-gene interactions that exemplify the complexity of this approach.

As we begin to move toward the concept of actionable intervention targets as an approach for implementing precision nutrition to reduce CVD risk, it is important to first recognize and appreciate the inherent complexity of human nutritional biochemistry. Unfortunately, it is not enough to simply genotype individuals in order to discover how genotype interacts with any given dietary component to modify phenotype. A compelling example is that of coffee intake. An exciting and relatable early success of nutrigenomics was the discovery that consuming > 2 cups of coffee per day was associated with increased blood pressure and as much as a 50% increase in myocardial infarction (MI) risk in individuals with the “slow caffeine metabolizer” *CYP1A2* genotype (rs2472300) [63]. This was a compelling finding, with a plausible explanation for biological mechanism, and a nice target for intervention. Rather than having to worry about the complexities of balancing micro- and macro-nutrient needs with food preparation and meal planning as is usually the case, in this instance we are simply looking at modifying whether and how much coffee an individual drinks. Of course, actually implementing this change and asking a patient to stop drinking (or significantly reduce) their coffee in the morning is not an easy task. Nonetheless, coffee is one dietary component that is easy to measure the intake of (i.e., individuals know whether they drink coffee or not and tend to have routines established around coffee intake that are consistent and reliable), and for which there are also excellent metabolomic markers in plasma and urine [64, 65].

Unfortunately, simply measuring *CYP1A2* genotype may not be adequate in and of itself to determine an individual’s risk for MI related to coffee intake. A subsequent study examining the relationship between coffee intake and CVD risk using data from the UK Biobank found higher CVD risk in individuals who drink no coffee, decaffeinated coffee, or those who drink > 6 cups/day, and no relationships between the *CYP1A2* genotype, coffee intake, and MI risk [66]. Although it is possible that the disagreement is due to differences in study design or statistical approaches, it is also highly likely that there are fundamental differences between the populations studied. The first study was conducted in Costa Rica, whereas the larger study used data from a UK population. There may be many differences between the Costa Rican and UK populations that could be contributing to the differences in the effects of coffee intake, from the type of coffee that is consumed, to the way coffee is prepared (e.g., filtered vs. not filtered), to the intake of specific foods and/or medications and supplements containing molecules that affect *CYP1A2* expression and/or activity, to the diversity of the populations and their cultural practices. Nutrition is complicated even with something as “simple” as caffeine which has a well-characterized metabolic pathway with genetic polymorphisms known to influence flux through this pathway, and relatively few sources of the food component (i.e., a relatively short list of foods and beverages contain caffeine compared to, for example, foods and beverages that contain carbohydrates). The CYP1A2 enzyme that metabolizes caffeine also metabolizes many additional substrates, including diet-derived molecules from foods and spices such as turmeric, peppermint, grapefruit, and chamomile as well as medications including certain antibiotics which decrease enzyme activity, and conversely components in broccoli, cabbage, and chargrilled meat which increase enzyme activity [65, 67]. Coffee also contains additional bioactive molecules besides caffeine, which may also contribute to modifying CVD risk [68].

Most aspects of human biochemistry are equally if not more complex. For targets such as coffee intake, the measurement of *CYP1A2* genotype is a first step, but assessing overall phenotype in addition to genotype is necessary. *N*-of-1 experiments involving the reduction in or cessation of caffeine intake (whether that be decreasing or substituting regular coffee intake for decaffeinated coffee, or with other non-caffeinated substitutes) and measurement of blood pressure, sleep quality, anxiety, and other quality of life metrics would greatly add to the assessment of whether an individual’s caffeine intake is contributing negatively to their overall CVD risk profile.

Egg intake is another example of a dietary component with potential impact on CVD risk, which has been highly controversial over the years [69•]. The potential issues with eggs for individuals at risk for CVD include both their high cholesterol content and their choline content. Initially, studies found a positive relationship between the intake of eggs and other high-cholesterol foods, and plasma cholesterol concentrations [70]. However, dietary cholesterol was recently found across multiple studies not to be linked with increases in plasma cholesterol in many individuals, and this observation led to the removal of dietary cholesterol restriction from the current USDA dietary guidelines [71]. Nonetheless, approximately 1 in 2.4 million and 1 in 360,000 of individuals hyper-absorb cholesterol due to loss-of-function polymorphisms in the ATP-binding cassette (ABC) G5 and ABCG8 transporters, respectively, which normally “spit out” cholesterol absorbed in the intestinal tract [72, 73], and thus can and do, in fact, experience increases in plasma cholesterol concentrations in response to dietary cholesterol intake. The population-wide recommendation not to worry about dietary cholesterol because it does not affect “most people” not only does not apply to the individual who hyper-absorbs dietary cholesterol, but in fact directly puts them at higher risk. The precision nutrition approach in this case would be to identify those individuals who are cholesterol hyper-absorbers by measuring plasma concentrations of plant sterols, which are increased in cholesterol hyper-absorbers [74], genetically screening for the *ABCG5/8* polymorphisms, and/or conducting an *n*-of-1 experiment in which a patient avoids all dietary sources of cholesterol for a fixed amount of time while keeping all other aspects of diet and lifestyle constant, and measuring lipid profiles before and after to determine whether LDL cholesterol or ApoB concentrations decreased [69•].

The other component of eggs that contributes to the confusion around egg intake is choline, because of its role as a precursor for the synthesis of trimethylamine-N-oxide (TMAO), a microbe-host co-metabolite whose concentrations have been found to be independently associated with CVD risk [75–77]. High circulating plasma TMAO concentrations were first discovered in individuals with CVD [78], and subsequently found to be associated with impaired reverse cholesterol transport and impairment of bile production [79, 80]. However, plasma TMAO concentrations have notoriously high inter-individual variability among the population, with two steps in the process being involved in mediating this variability. In the first step, gut microbiota convert choline to TMA in the gut [77]. In the second step, the absorbed TMA is further converted to TMAO in the liver [77]. Although the measurement of gut microbiome profiles could be a promising approach for determining disease risk for other outcomes, it may not be ideal for assessing TMA production. The bacterial genes for conversion of choline (as well as carnitine) to TMA are not simply concentrated in one bacterial genus but rather spread across multiple genera across phyla and can also be expressed or not expressed depending on many additional factors related to overall gut microbiome, thus making it difficult to use gut microbiome profiles to determine risk [69•]. Genotyping for the flavin-containing monooxygenase 3 (FMO3) enzyme which converts TMA to TMAO is also unlikely to be informative enough, given that this enzyme also metabolizes a wide array of additional substrates which have positive and negative effects on enzyme activity and expression [77, 81], much as is the case with CYP1A2 discussed previously. Instead, physicians can measure the concentrations of TMAO in plasma directly [82]. If a patient has high TMAO concentrations an *n*-of-1 experiment to eliminate the main sources of choline and carnitine (i.e., meat, eggs, and/or fish, which can contain TMAO) one at a time from the diet would be the most direct approach to determine whether TMAO concentration is related to a specific dietary component for that individual and could thus be a viable intervention target by which to reduce CVD risk.

## Potential of HDL as a Next Generation Suite of Precision Nutrition Targets

Thus far the discussion has been focused on relatively easy to understand nutritional intervention targets with relatively clear outcome measures. However, there is a critical player in the CVD risk discussion that has been perhaps more controversial and confusing than anything else: HDL. Although HDL are protective against a wide array of diseases, enabling some lucky humans to live very long, healthy lives [83], the search for HDL therapeutics has largely been a confusing enterprise marked by disappointing failures [84]. HDL particles are complex, heterogeneous, and dynamic, with a half-life of 4–5 days [85], and with as many as 16 different functional, compositional, and size/charge-based subclasses [86, 87] that confer a broad range of functions, from the modulation of immune cell activation [88], to the delivery of critical antiproteases to sites of injury [89], to as yet not well-understood interactions across the blood–brain-barrier [90, 91]. At their very small size (7–15 nm in diameter), they are notoriously difficult to work with and fall into “no man’s land”: they are the size of molecules yet they are colloidal particles. Technologies for analyzing and separating small molecules (e.g., RNA, proteins, metabolites) are built for working with homogeneous structures. On the other hand, technologies for analyzing, separating, and counting particles and cells, which match HDL compositional and physicochemical behavior and heterogeneity (e.g., flow cytometry, nanoparticle tracking analysis), are built for much larger particles (60 nm–micron in diameter). Thus, HDL particles, which may very well be part of nature’s primordial immune system, remain one of the most elusive biological nanoparticles. HDL are most well-known for their role in reverse cholesterol transport, but HDL particles also perform a wide array of other functions, from anti-oxidant, to immunomodulatory, to anti-proteolytic [92]. HDL particles also transport RNA and regulate cellular gene expression [93]. It was recently discovered that HDL particles are even necessary for extracellular vesicles (EV) function as a component of the EV protein corona, with important implications for EV-mediated inter-cellular signaling [94].

HDL particles are actively remodeled through exchange of lipids and proteins with other lipoprotein classes in the plasma compartment (e.g., through cholesterol ester transfer protein (CETP)) [95], as part of their uptake and resecretion by a variety of cell types [96], and through interactions with their cell surface receptors, all of which significantly affect their composition, size, and function. Small HDL and pre β-HDL that are not highly lipidated interact primarily with the ABCA1 receptor, whereas large mature HDL are thought to bind preferentially to the ABCG1 and scavenger receptor class B type 1 (SR-BI), resulting in substantial remodeling of the particles and differential cholesterol efflux capacity by particle subclass based on structure, size, and composition [86]. There is a body of literature showing that a variety of physiologically relevant chemical modifications can also modify HDL particle physicochemical characteristics. For example, oxidation, which can result from exposure to various oxidation sources including myeloperoxidase produced by neutrophils [97], can damage HDL proteins [98] and lipids [99], leading to both loss of beneficial function (e.g., cholesterol efflux) [99] and gain of deleterious function (e.g., pro-inflammatory effect on endothelial cells) [97]. In conditions where hyperglycemia is present, as in type 1 and type 2 diabetes, glycation of HDL impairs its cholesterol efflux capacity and ability to inhibit endothelial inflammation [100] and can even exacerbate cellular senescence [101]. On the other hand, exposure to low pH, as may be encountered in areas of hypoxia due to increased anaerobic metabolism and lactic acid production, increases both particle size and cholesterol efflux capacity [102].

In addition to the complexity already inherent in the well-characterized subfractions within the classically defined 1.063–1.21 g/mL HDL density range [103–106], it has been known since 1966 that HDL particles in the 1.21–1.25 g/mL density range can also be found [107], about which nothing is yet known. Proteomic analyses reveal that these particles have a unique proteome, harboring many of the functional proteins (e.g., CETP) related to some of the most compelling HDL functions including exchange of lipids and other molecules between lipoproteins, sequestration of lipopolysaccharides, and anti-oxidant function (unpublished data).

### Effects of Diets and Dietary Components on HDL Function and Composition

The effects of diets and nutritional factors on HDL particle composition and function have been studied and reviewed elsewhere [108]. Analysis from a subsample of the PREDIMED Study (Prevención con Dieta Mediterránea) with 296 subjects showed that compared to a low-fat diet, the groups with interventions of traditional Mediterranean diets enriched with virgin olive oil and enriched with nuts had improved HDL cholesterol efflux capacity (CEC); Mediterranean diet enriched with virgin olive oil specifically reduced CETP activity, improved lecithin-cholesterol acyltransferase (LCAT), paraoxonase-1, and vasodilatory capacities [109]. Standard DASH diet intervention lowered HDL-C, but did not change the concentration of small, medium, or large HDL [110], while no reports on the effects of the DASH diet on HDL functions have been found. Individual variability was a larger variable than dietary supplement in these study observations. With physical activity modification, improving diet quality by selecting lean sources of protein, unsaturated fat, and nutrient-dense food increased HDL CEC by 14% or 3.4%, depending on the cell model (J774 or HepG2, respectively) [111]. In general, the effects of dietary intervention appear to be modest in effect. For example, although extra virgin olive oil, which is enriched with lipid-soluble polyphenols, was found to increase HDL CEC, arguably the most well-studied of the HDL functional parameters, the increase in CEC was only about 2% compared to low-polyphenol olive oil [112]. Likewise, the increase in HDL CEC in response to the intake of whole eggs compared to egg whites was about 6% [113]. Interestingly, it appears that not eating may have the most potent effect on CEC, with a single bout of 36 h of water-only fasting increasing CEC by 20% compared to an overnight fast and 50% compared to the postprandial state [114]. The inter-individual variability in HDL response to different diets and dietary components has not been explicitly studied though in all trials cited above, for example, there is evidence of high inter-individual variability.

### Intestinally Derived HDL

An underappreciated observation is that a significant proportion of the circulating HDL pool is derived from the intestinal tract (approximately 30%) [115], and a significant proportion of the RNA transported by HDL particles is of bacterial origin [116]. Thus, there are likely as yet poorly understood interactions between HDL particles and the gut microbiome. In an examination of the relative contribution of the gut microbiome in modulating different plasma lipid profile parameters, emerging evidence indicates an interplay between signaling molecules generated by the gut microbiome and HDL particles that can strongly influence HDL function, such as their ability to efflux cholesterol, impacting CVD risk, as reviewed elsewhere [117]. For example, Koeth et al. showed that intestinal microbiota metabolism of l-carnitine, abundant in red meat, produces TMAO which accelerates atherosclerosis in mice and is associated with increased CVD risk in humans, while chronic l-carnitine supplementation in mice changes gut microbiota composition, increases TMAO synthesis, and reduces reverse cholesterol transport [118]. Given the evidence of these complex interactions between intestinal microbiota, HDL particles, and CVD risk, the significance of intestinally derived HDL in maintaining bodily homeostasis and preventing disease is becoming increasingly clear.

Dietary fiber intake, for instance, is known to enhance the diversity of gut microbial communities [119]. Many of these microbes perform essential roles such as producing metabolites crucial for immune function, maintaining gut barrier integrity, and modulating circulating endotoxins [120]. Despite these efforts, endotoxins can still make their way into the circulation [121]. A recent study sheds light on how intestinally derived HDL helps manage these endotoxins [122]. HDL particles, originating from the gut, were found to bind to lipopolysaccharide binding proteins on the basolateral side of the enterocytes [122]. They then directly enter the bloodstream via the capillary, moving into the portal vein. This journey plays a critical role in protecting the liver from excessive immune cell activation by sequestering these endotoxins [122]. Additionally, intestinally derived HDLs carry apolipoprotein A-IV (ApoA-IV), a multifunctional protein associated with protection against atherosclerotic CVD [123, 124]. A recent human metabolic tracer study revealed a unique, size-dependent metabolic behavior of ApoA-IV [125]. Once synthesized, ApoA-IV enters the lymphatic system where its subsequent release into the bloodstream depends on the size of the associated HDL particles [125]. Smaller HDL particles carrying ApoA-IV appeared in circulation approximately 30 min post-synthesis, followed by rapid catabolism, whereas larger HDL particles with ApoA-IV took longer to enter circulation, typically between 1 and 2 h, and demonstrated a slower rate of catabolism [125]. It is becoming increasingly evident that HDL are far more versatile than previously thought, and it is possible that intestinally derived HDL are equally complex, elusive, and critical to understand in disease prevention and biology as liver-derived particles. The first obstacle in the path to discovery is the fact that there are currently no practicable ways to specifically isolate intestinally derived HDL from the HDL pool in humans, thus very little is known about the structure, composition, and function of this class of HDL in human biology.

### HDL Glycosylation

Whereas it has been appreciated for some time that HDL particles are complex in part due to high heterogeneity in their protein and lipid composition, additional layers of complexity have emerged: HDL particles are also glycosylated [126]. The contribution of lipids and proteins to the size, structure, and function of HDL particles has been characterized [127, 128]. Yet the contribution of HDL glycosylation to HDL function and structure is in its infancy. So far it has it been shown that HDL particles are highly sialylated [126], that HDL glycosylation profiles can distinguish healthy individuals from those with disease [129], are correlated with HDL functional parameters [130, 131], and are differentially distributed across the lipoprotein fractions [132].

Protein glycosylation is a crucial intracellular enzymatic post-translational modification, influencing a protein’s functional three-dimensional configuration and contributing to its charge and various mechanisms, including trafficking and affinity to physiological targets [132, 133]. Assessing HDL glycosylation and its impact on function presents a challenging task, as it involves isolating particles while preserving their native configuration and glycan structures [134].

Given the significance of HDL glycosylation in its function, exploring therapeutic approaches to enhance function by modulating glycosylation is a captivating area of research [135]. Understanding the role of glycosylation in HDL particles could open new opportunities for improving their function and potentially benefitting human health by interventions such as diet and exercise. A compelling study comparing the effects of a Mediterranean diet to a fast-food diet demonstrated significant alterations in apolipoprotein C-III glycosylation, which were found to be correlated with changes in HDL function and immunomodulatory capacity [131]. This finding suggests that dietary patterns can influence HDL glycosylation, consequently impacting HDL’s protective functions and its role in immune regulation. Additionally, a study involving young children in Ghana showed that a lipid-rich supplement led to altered glycopeptides, which were also correlated with changes in HDL cholesterol efflux [136]. This observation implies that targeted dietary supplementation can potentially modify HDL glycosylation and subsequently improve its cholesterol transport capabilities, which are vital for cardiovascular health.

Despite being in its early stages, this area of research has promising implications regarding the interplay between dietary and lifestyle interventions with HDL glycosylation and its consequential impact on HDL function. Alterations in HDL glycosylation profiles have the potential to significantly influence HDL’s physiological roles, offering exciting prospects for personalized health interventions that could ultimately reduce the risk of CVD and other pathological conditions.

### Utility and Importance of Future Technologies That Enable the Measurement of Per Particle Composition and Function

The effects of the most influential genotypes on HDL metabolism have been well-characterized and described, including the effects of LCAT [137–139], apolipoprotein A-I (ApoA1) [140], and CETP deficiency [141, 142]. More recent work has demonstrated the impacts of *APOE* genotype on HDL-related outcomes. Whereas Alzheimer’s patients with the *APOE3/E4* genotype had smaller median HDL particle size, dementia patients with the *APOE3/E3* genotype had no difference in particle size but had decreased CEC and LCAT activity compared with their respective *APOE3/E3* genotype controls [143•]. Paradoxically, *APOE3/E4* dementia patients actually had higher HDL CEC than *APOE3/E4* controls [143•]. It was hypothesized that since isolated HDL particles were dosed by protein content in the CEC assay, for patients with higher relative proportions of smaller particles as much as twofold more particles may have been in each experimental well, resulting from the fact that small HDL particles have a higher protein:lipid ratio than larger particles. Indeed, when the average size of HDL particles was measured, the *APOE3/E4* but not the *APOE3/E3* dementia patients had smaller average HDL particle size than their respective controls [143•]. This raises a fundamentally important question about the contribution of HDL particle size distribution profile to the analysis of HDL composition and function.

We contend that in order for HDL compositional and functional analysis to be useful in terms of both understanding HDL biology and for the development of diagnostics and biomarkers, it will be necessary to fractionate HDL into size-based subclasses and also dose any compositional and functional assays by particle concentration. Without the ability to report, for example, the amount of ApoE molecules per particle or the CEC per particle, it will be impossible to disentangle the complexity of HDL biology. The influence of *APOE* genotype is an interesting illustration of this. As mentioned previously, *APOE3/E4* dementia patients had smaller median particle size but no difference in LCAT activity and a surprising increase in CEC compared to *APOE3/E4* controls [143•]. We also observed that individuals with *APOE3/E4* genotype had lower CEC and LCAT activity than individuals with *APOE3/E3* genotype regardless of dementia diagnosis [143•]. Further examination revealed that the HDL particle size distribution profiles of *APOE3/E4* dementia patients resembled those of individuals with type 2 diabetes (unpublished data), with higher concentrations of small HDL and lower concentrations of medium and large HDL [144]. This HDL particle size distribution profile of higher small HDL and lower medium and large HDL is most likely explained by the well-characterized effects of heightened CETP activity in the context of high plasma triglycerides [145]. Thus, metabolic status affects HDL particle size distribution profiles, as do other conditions, for example, chronic kidney disease, in which LCAT activity is decreased due to lack of ability to esterify cholesterol and thus form mature, larger particles [146, 147].

On the other hand, pathological processes of chronic disease states including inflammation, oxidation, exposure to high concentrations of glucose, and low pH, all physically damage HDL particles and decrease their functional capacity including CEC, antioxidant capacity, and anti-inflammatory function [102, 148–151]. Thus, in order to tease apart whether the underlying problem in any given individual is metabolic remodeling of HDL particle size distribution, or exposure to damaging chemicals that impair HDL function without affecting particle size, or a combination of both, it will be necessary to examine functional and compositional changes on a per particle basis within HDL size-based subclasses. Once specific targets for intervention are known (e.g., loss of antioxidant function of medium HDL, loss of large HDL particles, decrease in ApoE per small HDL particle), it will finally be possible to begin to work toward specific solutions.

## Improving Adherence with Precision Nutrition

Adherence poses a major challenge limiting the real-world impact of dietary recommendations on health outcomes [7]. However, emerging precision nutrition techniques may offer solutions to overcome this barrier. For example, by tailoring dietary advice to each individual based on genetics, such as *APOE* genotype, phenotype, and other personal data, recommendations can become more targeted and perceived as relevant by patients [152]. Genetic testing could identify patients with specific genetic polymorphisms influencing HDL function [143•], enabling personalized guidance to optimize their HDL profile. Additionally, using biomarker panels to evaluate diet quality may motivate individuals to improve compliance when they can see the impact of dietary changes reflected in their biomarker profile [153, 154]. Rather than eliminating foods or food groups, testing for individual sensitivities and gradually removing triggers based on measurable responses may promote better long-term adherence. While more research is still required, these types of personalized, feedback-driven precision nutrition approaches show promise for overcoming adherence issues that limit many one-size-fits-all dietary interventions. Overcoming these adherence challenges is essential to promote individualized recommendations into meaningful improvements in patient HDL status and CVD risk reduction.

## Conclusions

Precision nutrition approaches will revolutionize the ability of clinicians to determine risk and improve dietary recommendations for the individual patient with higher accuracy, leading to not only improved patient compliance but more importantly, improved health outcomes and the possibility of reducing CVD risk much earlier than is currently recommended. Although precision nutrition is still in its early stages, some dietary components (e.g., caffeine intake, dietary cholesterol intake) have already shown promise as targets for CVD risk modification in individuals. Beyond the management of population-wide intervention targets (e.g., management of blood pressure, lipid profiles), new precision nutrition intervention targets are needed to further decrease CVD risk in individuals. On the horizon are precision nutrition intervention targets aimed at ameliorating HDL particles.

## References

[1] Kaput J, Rodriguez RL (2006). Nutritional genomics: discovering the path to personalized nutrition.

[2] German JB, Zivkovic AM, Dallas DC, Smilowitz JT (2011). Nutrigenomics and personalized diets: what will they mean for food?. Annu Rev Food Sci Technol.

[3] De Toro-Martín J, Arsenault B, Després J-P, Vohl M-C (2017). Precision nutrition: a review of personalized nutritional approaches for the prevention and management of metabolic syndrome. Nutrients.

[4] Konstantinidou V, Ruiz LAD, Ordovás JM (2014). Personalized nutrition and cardiovascular disease prevention: from framingham to PREDIMED. Adv Nutr.

[5] Zhang Y-B, Pan X-F, Chen J, et al. Combined lifestyle factors, all-cause mortality and cardiovascular disease: a systematic review and meta-analysis of prospective cohort studies. J Epidemiol Community Health. 2020;jech-2020–214050. 10.1136/jech-2020-214050. 10.1136/jech-2020-214050 32892156

[6] AlAufi NS, Chan YM, Waly MI (2022). Application of Mediterranean diet in cardiovascular diseases and type 2 diabetes mellitus: motivations and challenges. Nutrients.

[7] De Mestral C, Khalatbari-Soltani S, Stringhini S, Marques-Vidal P (2020). Perceived barriers to healthy eating and adherence to dietary guidelines: nationwide study. Clin Nutr.

[8] Lichtenstein AH, Appel LJ, Vadiveloo M, et al. 2021 dietary guidance to improve cardiovascular health: a scientific statement from the American Heart Association. Circulation. 2021;144. 10.1161/CIR.0000000000001031. 10.1161/CIR.0000000000001031 34724806

[9] Bedsaul-Fryer JR, Van Zutphen-Küffer KG, Monroy-Gomez J (2023). Precision nutrition opportunities to help mitigate nutrition and health challenges in low- and middle-income countries: an expert opinion survey. Nutrients.

[10] Hand RK, Davis AM, Thompson KL (2021). Updates to the definition of evidence-based (dietetics) practice: providing clarity for practice. J Acad Nutr Diet.

[11] Wang L, Wang S, Zhang Q (2022). The role of the gut microbiota in health and cardiovascular diseases. Mol Biomed.

[12] Elijovich F, Laffer CL, Sahinoz M (2020). The gut microbiome, inflammation, and salt-sensitive hypertension. Curr Hypertens Rep.

[13] Kawarazaki W, Fujita T (2021). Kidney and epigenetic mechanisms of salt-sensitive hypertension. Nat Rev Nephrol.

[14] Zeevi D, Korem T, Zmora N (2015). Personalized nutrition by prediction of glycemic responses. Cell.

[15] Mazidi M, Valdes AM, Ordovas JM (2021). Meal-induced inflammation: postprandial insights from the Personalised REsponses to DIetary Composition Trial (PREDICT) study in 1000 participants. Am J Clin Nutr.

[16] Berry SE, Valdes AM, Drew DA (2020). Human postprandial responses to food and potential for precision nutrition. Nat Med.

[17] Griffin B, Walker C, Jebb S (2018). APOE4 genotype exerts greater benefit in lowering plasma cholesterol and apolipoprotein B than wild type (E3/E3), after replacement of dietary saturated fats with low glycaemic index carbohydrates. Nutrients.

[18] Zivkovic AM, Wiest MM, Nguyen U (2009). Assessing individual metabolic responsiveness to a lipid challenge using a targeted metabolomic approach. Metabolomics.

[19] Gross G, Jacobs DM, Peters S (2010). In vitro bioconversion of polyphenols from black tea and red wine/grape juice by human intestinal microbiota displays strong interindividual variability. J Agric Food Chem.

[20] Vega-López S, Ausman LM, Griffith JL, Lichtenstein AH (2007). Interindividual variability and intra-individual reproducibility of glycemic index values for commercial white bread. Diabetes Care.

[21] Sacks FM, Lichtenstein AH, Wu JHY, et al. Dietary fats and cardiovascular disease: a presidential advisory from the American Heart Association. Circulation. 2017;136. 10.1161/CIR.0000000000000510. 10.1161/CIR.0000000000000510 28620111

[22] Kris-Etherton PM (1999). Monounsaturated fatty acids and risk of cardiovascular disease. Circulation.

[23] Connelly MA, Otvos JD, Shalaurova I (2017). GlycA, a novel biomarker of systemic inflammation and cardiovascular disease risk. J Transl Med.

[24] Ballout RA, Remaley AT. GlycA: a new biomarker for systemic inflammation and cardiovascular disease (CVD) risk assessment. J Lab Precis Med. 2020;5:17–17. 10.21037/jlpm.2020.03.03. 10.21037/jlpm.2020.03.03 PMC7194207 32363327

[25] Brassard D, Tessier-Grenier M, Allaire J (2017). Comparison of the impact of SFAs from cheese and butter on cardiometabolic risk factors: a randomized controlled trial. Am J Clin Nutr.

[26] Getz G, Reardon C (2018). Apoprotein E and reverse cholesterol transport. Int J Mol Sci.

[27] Liu S, Liu J, Weng R (2019). Apolipoprotein E gene polymorphism and the risk of cardiovascular disease and type 2 diabetes. BMC Cardiovasc Disord.

[28] Elias-Sonnenschein LS, Viechtbauer W, Ramakers IHGB (2011). Predictive value of APOE-ε4 allele for progression from MCI to AD-type dementia: a meta-analysis. J Neurol Neurosurg Psychiatry.

[29] Shatwan IM, Weech M, Jackson KG (2017). Apolipoprotein E gene polymorphism modifies fasting total cholesterol concentrations in response to replacement of dietary saturated with monounsaturated fatty acids in adults at moderate cardiovascular disease risk. Lipids Health Dis.

[30] Carvalho-Wells AL, Jackson KG, Lockyer S (2012). APOE genotype influences triglyceride and C-reactive protein responses to altered dietary fat intake in UK adults. Am J Clin Nutr.

[31] Fallaize R, Celis-Morales C, Macready AL (2016). The effect of the apolipoprotein E genotype on response to personalized dietary advice intervention: findings from the Food4Me randomized controlled trial. Am J Clin Nutr.

[32] Whelton PK, Carey RM, Aronow WS (2018). 2017 ACC/AHA/AAPA/ABC/ACPM/AGS/APhA/ASH/ASPC/NMA/PCNA guideline for the prevention, detection, evaluation, and management of high blood pressure in adults: executive summary: a report of the American College of Cardiology/American Heart Association Task Force on Clinical Practice Guidelines. Hypertension.

[33] Virani SS, Newby LK, Arnold SV, et al. 2023 AHA/ACC/ACCP/ASPC/NLA/PCNA guideline for the management of patients with chronic coronary disease. J Am Coll Cardiol. 2023;S0735109723052816. 10.1016/j.jacc.2023.04.003.

[34] Jones DW (2004). Dietary sodium and blood pressure. Hypertension.

[35] Blaustein MP, Zhang J, Chen L, Hamilton BP (2006). How does salt retention raise blood pressure?. Am J Physiol-Regul Integr Comp Physiol.

[36] Ying W-Z, Sanders PW (2002). Increased dietary salt activates rat aortic endothelium. Hypertension.

[37] Agrotis A (2005). The genetic basis for altered blood vessel function in disease: large artery stiffening. Vasc Health Risk Manag.

[38] Sacks FM, Svetkey LP, Vollmer WM (2001). Effects on blood pressure of reduced dietary sodium and the dietary approaches to stop hypertension (DASH) diet. N Engl J Med.

[39] Li K, Huang T, Zheng J (2014). Effect of marine-derived n-3 polyunsaturated fatty acids on C-reactive protein, interleukin 6 and tumor necrosis factor α: a meta-analysis. PLoS ONE.

[40] Wu P-Y, Chen K-M, Tsai W-C (2021). The Mediterranean dietary pattern and inflammation in older adults: a systematic review and meta-analysis. Adv Nutr.

[41] Zivkovic AM, Telis N, German JB, Hammock BD (2011). Dietary omega-3 fatty acids aid in the modulation of inflammation and metabolic health. Calif Agric.

[42] Serini S, Calviello G (2020). Omega-3 PUFA responders and non-responders and the prevention of lipid dysmetabolism and related diseases. Nutrients.

[43] Burdge GC, Calder PC (2006). Dietary α-linolenic acid and health-related outcomes: a metabolic perspective. Nutr Res Rev.

[44] Nording ML, Yang J, Georgi K (2013). Individual variation in lipidomic profiles of healthy subjects in response to omega-3 fatty acids. PLoS ONE.

[45] Zivkovic AM, Yang J, Georgi K (2012). Serum oxylipin profiles in IgA nephropathy patients reflect kidney functional alterations. Metabolomics Off J Metabolomic Soc.

[46] Vallée Marcotte B, Guénard F, Lemieux S (2019). Fine mapping of genome-wide association study signals to identify genetic markers of the plasma triglyceride response to an omega-3 fatty acid supplementation. Am J Clin Nutr.

[47] Rudkowska I, Guénard F, Julien P (2014). Genome-wide association study of the plasma triglyceride response to an n-3 polyunsaturated fatty acid supplementation. J Lipid Res.

[48] Chilton FH, Manichaikul A, Yang C (2022). Interpreting clinical trials with omega-3 supplements in the context of ancestry and FADS genetic variation. Front Nutr.

[49] King DE (2007). Effect of a high-fiber diet vs a fiber-supplemented diet on C-reactive protein level. Arch Intern Med.

[50] Ma W, Nguyen LH, Song M (2021). Dietary fiber intake, the gut microbiome, and chronic systemic inflammation in a cohort of adult men. Genome Med.

[51] Zhu C, Sawrey-Kubicek L, Beals E (2020). Human gut microbiome composition and tryptophan metabolites were changed differently by fast food and Mediterranean diet in 4 days: a pilot study. Nutr Res.

[52] David LA, Maurice CF, Carmody RN (2014). Diet rapidly and reproducibly alters the human gut microbiome. Nature.

[53] McPherson R, Tybjaerg-Hansen A (2016). Genetics of coronary artery disease. Circ Res.

[54] Fawcett KA, Barroso I (2010). The genetics of obesity: FTO leads the way. Trends Genet TIG.

[55] Kamar A, Khalil A, Nemer G (2021). The digenic causality in familial hypercholesterolemia: revising the genotype–phenotype correlations of the disease. Front Genet.

[56] Lambert CT, Sandesara P, Isiadinso I, et al. Current treatment of familial hypercholesterolaemia. Eur Cardiol. 2014;9:76–81. 10.15420/ecr.2014.9.2.76. 10.15420/ecr.2014.9.2.76 PMC6159444 30310490

[57] Hinney A, Vogel CIG, Hebebrand J (2010). From monogenic to polygenic obesity: recent advances. Eur Child Adolesc Psychiatry.

[58] Gustavsson J, Mehlig K, Leander K (2014). FTO genotype, physical activity, and coronary heart disease risk in Swedish men and women. Circ Cardiovasc Genet.

[59] Lan N, Lu Y, Zhang Y (2020). FTO – a common genetic basis for obesity and cancer. Front Genet.

[60] Grant SFA, Li M, Bradfield JP (2008). Association analysis of the FTO gene with obesity in children of Caucasian and African ancestry reveals a common tagging SNP. PLoS ONE.

[61] Ramos-Lopez O, Milagro FI, Allayee H (2017). Guide for current nutrigenetic, nutrigenomic, and nutriepigenetic approaches for precision nutrition involving the prevention and management of chronic diseases associated with obesity. Lifestyle Genomics.

[62] Kilpeläinen TO, Qi L, Brage S (2011). Physical activity attenuates the influence of FTO variants on obesity risk: a meta-analysis of 218,166 adults and 19,268 children. PLoS Med.

[63] Cornelis MC, El-Sohemy A, Kabagambe EK, Campos H (2006). Coffee, CYP1A2 genotype, and risk of myocardial infarction. JAMA.

[64] Papandreou C, Hernández-Alonso P, Bulló M (2019). Plasma metabolites associated with coffee consumption: a metabolomic approach within the PREDIMED study. Nutrients.

[65] Peterson S, Schwarz Y, Li SS (2009). *CYP1A2*, *GSTM1*, and *GSTT1* polymorphisms and diet effects on CYP1A2 activity in a crossover feeding trial. Cancer Epidemiol Biomarkers Prev.

[66] Zhou A, Hyppönen E (2019). Long-term coffee consumption, caffeine metabolism genetics, and risk of cardiovascular disease: a prospective analysis of up to 347,077 individuals and 8368 cases. Am J Clin Nutr.

[67] Chen Y, Liu W-H, Chen B-L (2010). Plant polyphenol curcumin significantly affects CYPIA2 and CYP2A6 activity in healthy, male Chinese volunteers. Ann Pharmacother.

[68] Frost-Meyer NJ, Logomarsino JV (2012). Impact of coffee components on inflammatory markers: a review. J Funct Foods.

[69] • Kang JW, Zivkovic AM. Are eggs good again? A precision nutrition perspective on the effects of eggs on cardiovascular risk, taking into account plasma lipid profiles and TMAO. J Nutr Biochem. 2022;100:108906. 10.1016/j.jnutbio.2021.108906. **This perspective paper discusses the evidence for the need for a precision health approach in determining the suitability of egg consumption for patients at risk for cardiovascular disease and discusses how to evaluate the suitability of egg intake for the individual patient. The discussion takes into account both the cholesterol and choline content of eggs and thus addresses both the effects of eggs on plasma lipid profiles via cholesterol and on plasma concentrations of trimethylamine N-oxide (a bacterially derived metabolite) via choline.** 10.1016/j.jnutbio.2021.108906 34801688

[70] Roberts SL, McMurry MP, Connor WE (1981). Does egg feeding (i.e., dietary cholesterol) affect plasma cholesterol levels in humans? The results of a double-blind study. Am J Clin Nutr.

[71] Millen BE, Abrams S, Adams-Campbell L (2016). The 2015 Dietary Guidelines Advisory Committee scientific report: development and major conclusions. Adv Nutr.

[72] Gylling H, Hallikainen M, Pihlajamäki J (2004). Polymorphisms in the ABCG5 and ABCG8 genes associate with cholesterol absorption and insulin sensitivity. J Lipid Res.

[73] Hooper AJ, Bell DA, Hegele RA, Burnett JR (2017). Clinical utility gene card for: sitosterolaemia. Eur J Hum Genet.

[74] Matthan NR, Resteghini N, Robertson M (2010). Cholesterol absorption and synthesis markers in individuals with and without a CHD event during pravastatin therapy: insights from the PROSPER trial. J Lipid Res.

[75] DiMarco DM, Missimer A, Murillo AG (2017). Intake of up to 3 eggs/day increases HDL cholesterol and plasma choline while plasma trimethylamine-N-oxide is unchanged in a healthy population. Lipids.

[76] Zhu C, Sawrey-Kubicek L, Bardagjy AS (2020). Whole egg consumption increases plasma choline and betaine without affecting TMAO levels or gut microbiome in overweight postmenopausal women. Nutr Res.

[77] Bennett BJ, Vallim TQ de A, Wang Z, et al. Trimethylamine-N-oxide, a metabolite associated with atherosclerosis, exhibits complex genetic and dietary regulation. Cell Metab. 2013;17:49–60. 10.1016/j.cmet.2012.12.011. 10.1016/j.cmet.2012.12.011 PMC3771112 23312283

[78] Brown JM, Hazen SL (2014). Metaorganismal nutrient metabolism as a basis of cardiovascular disease. Curr Opin Lipidol.

[79] Ding L, Chang M, Guo Y (2018). Trimethylamine-N-oxide (TMAO)-induced atherosclerosis is associated with bile acid metabolism. Lipids Health Dis.

[80] Wang Z, Klipfell E, Bennett BJ (2011). Gut flora metabolism of phosphatidylcholine promotes cardiovascular disease. Nature.

[81] Esposito T, Varriale B, D’Angelo R, et al. Regulation of flavin-containing mono-oxygenase (Fmo3) gene expression by steroids in mice and humans. Horm Mol Biol Clin Investig. 2014;20. 10.1515/hmbci-2014-0012. 10.1515/hmbci-2014-0012 25460299

[82] Wang Z, Levison BS, Hazen JE (2014). Measurement of trimethylamine-N-oxide by stable isotope dilution liquid chromatography tandem mass spectrometry. Anal Biochem.

[83] Milman S, Atzmon G, Crandall J, Barzilai N (2013). Phenotypes and genotypes of high density lipoprotein cholesterol in exceptional longevity. Curr Vasc Pharmacol.

[84] Rosenson RS (2016). The high-density lipoprotein puzzle: why classic epidemiology, genetic epidemiology, and clinical trials conflict?. Arterioscler Thromb Vasc Biol.

[85] Blum CB, Levy RI, Eisenberg S (1977). High density lipoprotein metabolism in man. J Clin Invest.

[86] Kontush A, Lindahl M, Lhomme M, et al. Structure of HDL: particle subclasses and molecular components. In: Von Eckardstein A, Kardassis D, et al., editors. High density lipoproteins. Cham: Springer International Publishing; 2015. p. 3–51. 10.1007/978-3-319-09665-0_1 25522985

[87] Furtado JD, Yamamoto R, Melchior JT (2018). Distinct proteomic signatures in 16 HDL (high-density lipoprotein) subspecies. Arterioscler Thromb Vasc Biol.

[88] Bonacina F, Pirillo A, Catapano AL, Norata GD (2019). Cholesterol membrane content has a ubiquitous evolutionary function in immune cell activation: the role of HDL. Curr Opin Lipidol.

[89] ScottM G, McKenzie B, Kemeh G (2015). Rosuvastatin alters the proteome of high density lipoproteins: generation of alpha-1-antitrypsin enriched particles with anti-inflammatory properties*. Mol Cell Proteomics.

[90] Meilhac O, Von Eckardstein A, Kardassis D (2015). High-density lipoproteins in stroke. High density lipoproteins.

[91] Robert J, Stukas S, Button E (2016). Reconstituted high-density lipoproteins acutely reduce soluble brain Aβ levels in symptomatic APP/PS1 mice. Biochim Biophys Acta BBA - Mol Basis Dis.

[92] Rohatgi A, Westerterp M, von Eckardstein A (2021). HDL in the 21st century: a multifunctional roadmap for future HDL research. Circulation.

[93] Vickers KC, Michell DL (2021). HDL-small RNA export, transport, and functional delivery in atherosclerosis. Curr Atheroscler Rep.

[94] Liu K, Nilsson R, Lázaro-Ibáñez E (2023). Multiomics analysis of naturally efficacious lipid nanoparticle coronas reveals high-density lipoprotein is necessary for their function. Nat Commun.

[95] Tall A (1995). Plasma lipid transfer proteins. Annu Rev Biochem.

[96] Röhrl C, Stangl H (2013). HDL endocytosis and resecretion. Biochim Biophys Acta BBA - Mol Cell Biol Lipids.

[97] Undurti A, Huang Y, Lupica JA (2009). Modification of high density lipoprotein by myeloperoxidase generates a pro-inflammatory particle. J Biol Chem.

[98] Greilberger J, Jürgens G (1998). Oxidation of high-density lipoprotein HDL3 leads to exposure of apo-AI and apo-AII epitopes and to formation of aldehyde protein adducts, and influences binding of oxidized low-density lipoprotein to type I and type III collagen in vitro. Biochem J.

[99] Nagano Y, Arai H, Kita T (1991). High density lipoprotein loses its effect to stimulate efflux of cholesterol from foam cells after oxidative modification. Proc Natl Acad Sci.

[100] Hoang A, Murphy AJ, Coughlan MT (2007). Advanced glycation of apolipoprotein A-I impairs its anti-atherogenic properties. Diabetologia.

[101] Park K-H, Cho K-H (2011). High-density lipoprotein (HDL) from elderly and reconstituted HDL containing glycated apolipoproteins A-I share proatherosclerotic and prosenescent properties with increased cholesterol influx. J Gerontol A Biol Sci Med Sci.

[102] Nguyen SD, Öörni K, Lee-Rueckert M (2012). Spontaneous remodeling of HDL particles at acidic pH enhances their capacity to induce cholesterol efflux from human macrophage foam cells. J Lipid Res.

[103] Rosenson RS, Brewer HB, Chapman MJ (2011). HDL measures, particle heterogeneity, proposed nomenclature, and relation to atherosclerotic cardiovascular events. Clin Chem.

[104] Havel RJ, Eder HA, Bragdon JH (1955). The distribution and chemical composition of ultracentrifugally separated lipoproteins in human serum. J Clin Invest.

[105] Blanche PJ, Gong EL, Forte TM, Nichols AV (1981). Characterization of human high-density lipoproteins by gradient gel electrophoresis. Biochim Biophys Acta BBA - Lipids Lipid Metab.

[106] Alaupovic P, Lee DM, McConathy WJ (1972). Studies on the composition and structure of plasma lipoproteins. Biochim Biophys Acta BBA - Lipids Lipid Metab.

[107] Alaupovic P, Sanbar SS, Furman RH (1966). Studies of the composition and structure of serum lipoproteins. Isolation and characterization of very high density lipoproteins of human serum. Biochemistry.

[108] Bardagjy AS, Steinberg FM (2019). Relationship between HDL functional characteristics and cardiovascular health and potential impact of dietary patterns: a narrative review. Nutrients.

[109] Hernáez Á, Castañer O, Elosua R (2017). Mediterranean diet improves high-density lipoprotein function in high-cardiovascular-risk individuals: a randomized controlled trial. Circulation.

[110] Chiu S, Bergeron N, Williams PT (2016). Comparison of the DASH (dietary approaches to stop hypertension) diet and a higher-fat DASH diet on blood pressure and lipids and lipoproteins: a randomized controlled trial. Am J Clin Nutr.

[111] Boyer M, Mitchell PL, Poirier P (2018). Impact of a one-year lifestyle modification program on cholesterol efflux capacities in men with abdominal obesity and dyslipidemia. Am J Physiol-Endocrinol Metab.

[112] Hernáez Á, Fernández-Castillejo S, Farràs M (2014). Olive oil polyphenols enhance high-density lipoprotein function in humans: a randomized controlled trial. Arterioscler Thromb Vasc Biol.

[113] Sawrey-Kubicek L, Zhu C, Bardagjy AS (2019). Whole egg consumption compared with yolk-free egg increases the cholesterol efflux capacity of high-density lipoproteins in overweight, postmenopausal women. Am J Clin Nutr.

[114] Rhodes CH, Zhu C, Agus J (2022). Human fasting modulates macrophage function and upregulates multiple bioactive metabolites that extend lifespan in Caenorhabditis elegans: a pilot clinical study. Am J Clin Nutr.

[115] Brunham LR (2006). Intestinal ABCA1 directly contributes to HDL biogenesis in vivo. J Clin Invest.

[116] Allen RM, Zhao S, Ramirez Solano MA (2018). Bioinformatic analysis of endogenous and exogenous small RNAs on lipoproteins. J Extracell Vesicles.

[117] Nakaya K, Ikewaki K (2018). Microbiota and HDL metabolism. Curr Opin Lipidol.

[118] Koeth RA, Wang Z, Levison BS (2013). Intestinal microbiota metabolism of l-carnitine, a nutrient in red meat, promotes atherosclerosis. Nat Med.

[119] Cronin P, Joyce SA, O’Toole PW, O’Connor EM (2021). Dietary fibre modulates the gut microbiota. Nutrients.

[120] Wu H-J, Wu E (2012). The role of gut microbiota in immune homeostasis and autoimmunity. Gut Microbes.

[121] Kang JW, Tang X, Walton CJ, Brown MJ, Brewer RA, Maddela RL, et al. Multi-omic analyses reveal bifidogenic effect and metabolomic shifts in healthy human cohort supplemented with a prebiotic dietary fiber Blend. Front Nutr. 2022;9:908534. 10.3389/fnut.2022.908534. 10.3389/fnut.2022.908534 PMC9248813 35782954

[122] Han Y-H, Onufer EJ, Huang L-H, et al. Enterically derived high-density lipoprotein restrains liver injury via the portal vein. Science. 2021;373:eabe6729. 10.1126/science.abe6729. 10.1126/science.abe6729 PMC8478306 34437091

[123] Qu J, Ko C-W, Tso P, Bhargava A (2019). Apolipoprotein A-IV: a multifunctional protein involved in protection against atherosclerosis and diabetes. Cells.

[124] Peng J, Li X-P (2018). Apolipoprotein A-IV: a potential therapeutic target for atherosclerosis. Prostaglandins Other Lipid Mediat.

[125] Andraski AB, Singh SA, Higashi H (2023). The distinct metabolism between large and small HDL indicates unique origins of human apolipoprotein A4. JCI Insight.

[126] Huang J, Lee H, Zivkovic AM (2014). Glycomic analysis of high density lipoprotein shows a highly sialylated particle. J Proteome Res.

[127] Asztalos BF, Tani M, Schaefer EJ (2011). Metabolic and functional relevance of HDL subspecies. Curr Opin Lipidol.

[128] Li H, Gordon SM, Zhu X (2015). Network-based analysis on orthogonal separation of human plasma uncovers distinct high density lipoprotein complexes. J Proteome Res.

[129] Krishnan S, Huang J, Lee H (2015). Combined high-density lipoprotein proteomic and glycomic profiles in patients at risk for coronary artery disease. J Proteome Res.

[130] Krishnan S, Shimoda M, Sacchi R (2017). HDL glycoprotein composition and site-specific glycosylation differentiates between clinical groups and affects IL-6 secretion in lipopolysaccharide-stimulated monocytes. Sci Rep.

[131] Zhu C, Wong M, Li Q (2019). Site-specific glycoprofiles of HDL-associated ApoE are correlated with HDL functional capacity and unaffected by short-term diet. J Proteome Res.

[132] Kailemia MJ, Wei W, Nguyen K (2018). Targeted measurements of O- and N-glycopeptides show that proteins in high density lipoprotein particles are enriched with specific glycosylation compared to plasma. J Proteome Res.

[133] Maverakis E, Kim K, Shimoda M (2015). Glycans in the immune system and the altered glycan theory of autoimmunity: a critical review. J Autoimmun.

[134] Zheng JJ, Agus JK, Hong BV (2021). Isolation of HDL by sequential flotation ultracentrifugation followed by size exclusion chromatography reveals size-based enrichment of HDL-associated proteins. Sci Rep.

[135] Romo EZ, Zivkovic AM. Glycosylation of HDL-associated proteins and its implications in cardiovascular disease diagnosis, metabolism and function. Front Cardiovasc Med 2022;9:928566. 10.3389/fcvm.2022.928566. 10.3389/fcvm.2022.928566 PMC9184513 35694676

[136] Hong BV, Zhu C, Wong M (2021). Lipid-based nutrient supplementation increases high-density lipoprotein (HDL) cholesterol efflux capacity and is associated with changes in the HDL glycoproteome in children. ACS Omega.

[137] Asztalos BF, Schaefer EJ, Horvath KV (2007). Role of LCAT in HDL remodeling: investigation of LCAT deficiency states. J Lipid Res.

[138] Tietjen I, Hovingh GK, Singaraja R (2012). Increased risk of coronary artery disease in Caucasians with extremely low HDL cholesterol due to mutations in ABCA1, APOA1, and LCAT. Biochim Biophys Acta BBA - Mol Cell Biol Lipids.

[139] Vargas-Alarcon G, Perez-Mendez O, Herrera-Maya G (2018). *CETP* and *LCAT* gene polymorphisms are associated with high-density lipoprotein subclasses and acute coronary syndrome. Lipids.

[140] Koukos G, Chroni A, Duka A (2007). Naturally occurring and bioengineered apoA-I mutations that inhibit the conversion of discoidal to spherical HDL: the abnormal HDL phenotypes can be corrected by treatment with LCAT. Biochem J.

[141] Barkowski RS, Frishman WH (2008). HDL metabolism and CETP inhibition. Cardiol Rev.

[142] J. Niesor E, Von Der Mark E, Calabresi L,  (2012). Lipid and apoprotein composition of HDL in partial or complete CETP deficiency. Curr Vasc Pharmacol.

[143] • Hong BV, Zheng J, Agus JK, et al. High-density lipoprotein changes in Alzheimer’s disease are APOE genotype-specific. Biomedicines. 2022;10:1495. 10.3390/biomedicines10071495. **This study raises a fundamentally important question about the need to analyze HDL by particle size distribution profile and measure particle concentration, in addition to composition and function, to fully understand the complex biology of HDL subclasses.** 10.3390/biomedicines10071495 PMC9312991 35884800

[144] Sokooti S, Flores-Guerrero JL, Kieneker LM (2021). HDL particle subspecies and their association with incident type 2 diabetes: the PREVEND study. J Clin Endocrinol Metab.

[145] Murakami T, Michelagnoli S, Longhi R (1995). Triglycerides are major determinants of cholesterol esterification/transfer and HDL remodeling in human plasma. Arterioscler Thromb Vasc Biol.

[146] Yamatani K, Hirayama S, Seino U (2020). Preβ1-high-density lipoprotein metabolism is delayed in patients with chronic kidney disease not on hemodialysis. J Clin Lipidol.

[147] Gliwińska A, Ćwiklińska A, Czaplińska M (2022). Changes in the size and electrophoretic mobility of HDL subpopulation particles in chronic kidney disease. J Nephrol.

[148] Su X, Zhang G, Cheng Y, Wang B (2021). New insights into the emerging effects of inflammatory response on HDL particles structure and function. Mol Biol Rep.

[149] Fadaei R, Davies SS (2022). Oxidative modification of HDL by lipid aldehydes impacts HDL function. Arch Biochem Biophys.

[150] Cho K-H, Kim J-R, Lee I-C, Kwon H-J (2021). Native high-density lipoproteins (HDL) with higher paraoxonase exerts a potent antiviral effect against SARS-CoV-2 (COVID-19), while glycated HDL lost the antiviral activity. Antioxidants.

[151] Lee-Rueckert M, Lappalainen J, Leinonen H (2010). Acidic extracellular environments strongly impair ABCA1-mediated cholesterol efflux from human macrophage foam cells. Arterioscler Thromb Vasc Biol.

[152] Livingstone KM, Celis-Morales C, Navas-Carretero S (2021). Personalised nutrition advice reduces intake of discretionary foods and beverages: findings from the Food4Me randomised controlled trial. Int J Behav Nutr Phys Act.

[153] Hidalgo-Liberona N, Meroño T, Zamora-Ros R (2021). Adherence to the Mediterranean diet assessed by a novel dietary biomarker score and mortality in older adults: the InCHIANTI cohort study. BMC Med.

[154] Tong TYN, Koulman A, Griffin JL (2020). A combination of metabolites predicts adherence to the Mediterranean diet pattern and its associations with insulin sensitivity and lipid homeostasis in the general population: the Fenland study, United Kingdom. J Nutr.

